# WCM: A web content‐based method of stakeholder analysis

**DOI:** 10.1016/j.mex.2022.101635

**Published:** 2022-02-16

**Authors:** Susanne Raum, Felicity Rawlings-Sanaei

**Affiliations:** a Chair of Strategic Landscape Planning and Management, Technical University Munich, Germany / Imperial College London, UK; b Independent Researcher / Currently employed at Australian Catholic University, Australia

**Keywords:** Stakeholder mapping, Systematic stakeholder analysis, Qualitative research, Content analysis, Key word search

## Abstract

This article presents a systematic method to conduct stakeholder analyses using textual data from stakeholder websites. WCM, a novel web content-based method comprises stakeholder information and the use of keywords in a content analysis of relevant preselected stakeholder websites. Traditional stakeholder analysis approaches frequently rely on personal interpretation rather than empirical analysis. WCM aims to address this limitation by offering a user-friendly and empirical method that helps generate knowledge from multiple websites. With qualitative and / or quantitative application, it is particularly useful for small-scale studies in complex contexts and where resources are limited. WCM differs from many commonly used stakeholder analysis methods as it adopts replicable, systematic and transparent procedures. While not without limitations, this method provides an effective tool to support researchers, non-governmental organizations, and industry in different fields and locations, to undertake stakeholder analysis.

• The WCM method is a systematic, explicit, transparent, and transferable procedure to conduct stakeholder analysis.
• WCM has application in both qualitative and quantitative content analysis of stakeholder websites.
• WCM provides a user-friendly method to provide a broad overview of stakeholder interests with limited resources.

## Graphical abstract

**Figure:**
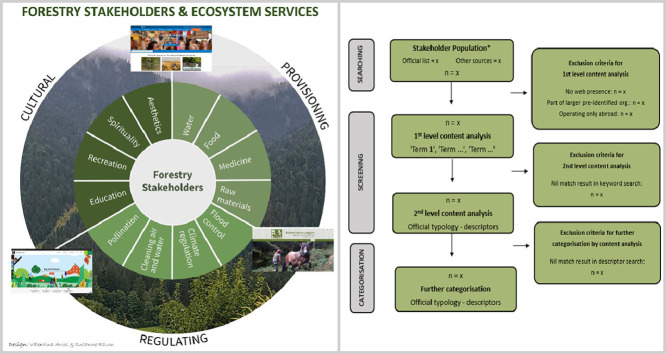

Specifications table

**Table:** 

Subject Area:	Social Science, Natural Resource Management, Environmental Science
More specific subject area:	*Stakeholder analysis in social and environmental sciences*
Method name:	*Web content-based method (WCM)*
Name and reference of original method:	*Evidence-based stakeholder categorization*[2]*A web content-based method of stakeholder analysis*[11]
Resource availability:	*The dataset on stakeholders in UK forestry and forest ecosystem services is publicly available on:* *https://doi.org/10.1016/j.jenvman.2021.113733* *https://www-sciencedirect-com.iclibezp1.cc.ic.ac.uk/science/article/pii/S0301479721017953*

## Rationale

A number of approaches have been developed to glean a deeper understanding of stakeholders in diverse settings [5,7]. One such approach, stakeholder analysis, offers a range of tools to identify and assess stakeholder activities [12]. Yet, comprehensive empirical stakeholder analysis can be heavily reliant upon resources and long-drawn out, frequently based on the investigator's personal interpretation rather than empirical analysis [2]. To address this limitation, a web content-based method (WCM) is proposed for the identification, verification, and categorization of stakeholders. User-friendly and novel, this empirical method examines stakeholder information on preselected stakeholder websites by means of keywords in a content analysis [11]. WCM differs from many commonly used stakeholder analysis methods as it adopts transferable, systematic and transparent procedures.

Websites often contain a wealth of information - both qualitative and quantitative - on an organization's objectives, strategy, products and markets [3]. They may also be a source of information on the interests of individual stakeholders. A plethora of websites, however, run to scores of pages of text, graphic, audio, and statistical information [3]. Using the abundant and varied data found on websites for stakeholder analyses, therefore, requires a strategic approach. Most importantly, researchers need to set clear boundaries within the context of the research [13] when conducting a content analysis [8] of stakeholder websites. This can be achieved through setting clear study objectives and the selection of clearly defined key words for the coding of textual content [6]. In this short article, we provide a technical description of the application of WCM for the systematic empirical identification, verification, and categorization of stakeholders [11]. Drawing on the WCM undertaken by Raum et al [11]. on stakeholders in UK forestry and forest ecosystem services, each step of the method is explained in detail in the following subsections.

## Method details

### Content analysis

Content analysis is a frequently applied social science method, designed to identify and document the views, attitudes, and interests of individuals, organizations, and diverse cultural groups of varying sizes [8]. Data used for this type of analysis can be existing or newly collected, i.e. secondary or primary data, in the form of texts stemming from a range of sources, including websites, documents, newspaper articles, recorded interviews, and social media text [1]. Three of the most common approaches to content analysis are as follows: (1) basic content analysis, which employs statistical analyses; (2) qualitative content analysis; and (3) interpretive content analysis. In an attempt to be systematic, objective, and transparent, basic content analyses use quantitative analytical methods, such as word counts, to analyze data. Data coding mainly consists of deductively or inductively generated code lists, typically involving existing texts [1]. Qualitative and interpretive content analyses, on the other hand, draw on narrative analysis methods, “describing the meaning of communications, in specific contexts, rather than on using quantitative word counts” ([1]: 2). In the WCM case study example, a basic content analysis approach was adapted to identify key words in existing textual data.

### Pre‐selection and verification of stakeholders

For smaller scale stakeholder analyses, especially if conducted on limited resources, existing stakeholder lists can be a useful starting point for analysis. Such lists, however, may not fit the exact purpose of the analysis and / or may be inaccurate. To avoid a large exclusion rate, it is therefore important to use a comprehensive and up-to-date list. Ideally, such a list should be obtained from an authoritative source and be closely linked to the research objectives and context. Alternatively, where such a list is unavailable, one could combine several stakeholder lists from a number of authoritative sources. For the web-based content analysis case study example, a list of 130 stakeholders compiled by the Forestry Commission [4] - the official UK Government Agency responsible for woodlands - was used. Further stakeholders were iteratively added to the original data collection (2013-2014) from other sources (literature, reports, websites), and revised in 2018 [11]. This approach resulted in a preliminary dataset of 175 stakeholders, comprising a broad range of businesses, industry, and not-for-profit and governmental organizations. The definition of ‘stakeholder’ was defined as ‘any organization, group, or individual interested in or with an influence over forests and forest ecosystem services based in the UK' [11].

The internet presence of each of these 175 stakeholders was then verified to ensure that they were still extant; were independent UK stakeholders; and did not form part of a pre-identified organization. Thirty-five of the 175 stakeholders were subsequently excluded [11]. After this initial data screening, two levels of content analysis of stakeholders’ respective websites were undertaken with the remaining 135 stakeholders. The first was to ascertain the general interest of these stakeholders in UK forests; the second was to determine their specific interests in UK forests (in terms of ecosystem services). At this juncture, it is important to note that, depending on the type of research questions, studies can have either one or multiple levels of content analyses. However, it is essential that for each level, clear boundaries are set.

### Selecting textual data and keywords for content analysis

**Fig. 1:**
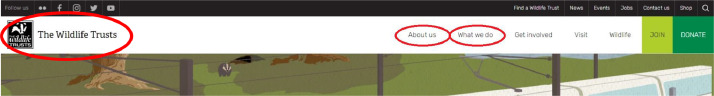
Screenshot example of homepage.

Since many websites contain large numbers of webpages, the collection of keyword data in this particular case study application of WCM was limited to three specific webpages: 1) ‘home’, 2) ‘about us’, and 3) ‘what we do’ (or equivalent) pages (Fig. 1). Based on a preliminary scoping review of website content, it was determined that these three webpages were the most likely to contain information on stakeholder aims, objectives and interests [11]. The keywords / descriptors used for the content analysis were derived from established typologies, as outlined below.

#### Content analysis level 1

**Fig. 2:**
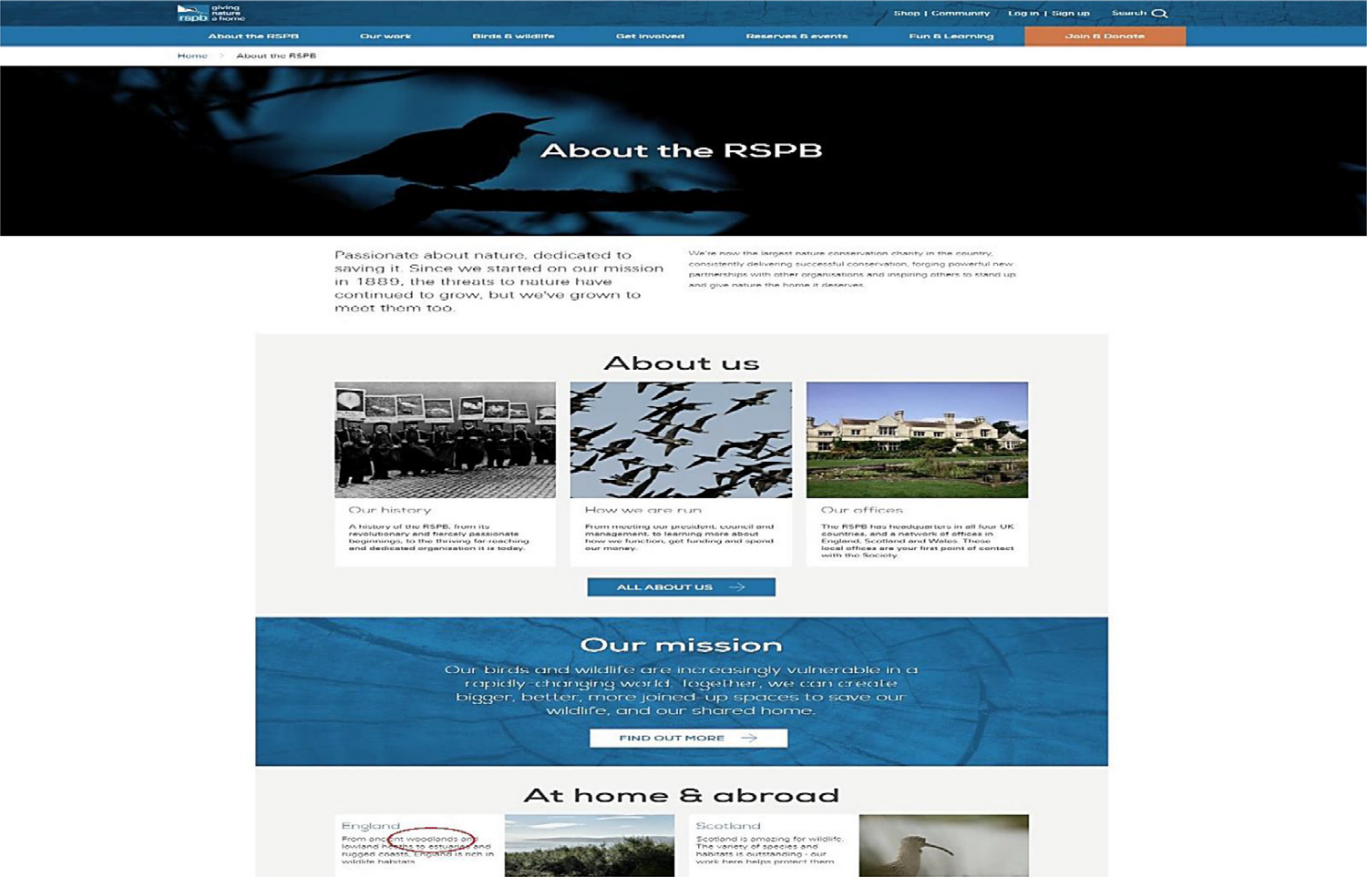
Screenshot example of ‘About Us’ webpage, mentioning woodland.

In this first phase, content analysis of stakeholder websites was undertaken to ascertain the interests of each of the 135 stakeholders in UK forests. Web content was searched using the search-key to scan for keywords ‘forest(s)’, ‘woodland(s)’ and ‘wood(s)’ and deductively coded with pre-determined codes for the keywords (Fig. 2). This served to filter out those stakeholders listed in the preliminary dataset who did not appear to have an explicit interest in UK forests, woods or woodlands (Raum et al., 2019). This also facilitated a more sophisticated analysis of the data and a second layer of analysis.

Since the frequency of the occurrence of the keywords had no bearing on this particular analysis (other than for exclusion), the word counts were irrelevant. Furthermore, there was no differential weighting of keywords as weighting also had no bearing on the analysis. The exclusion parameter was therefore strictly a nil match result in the keyword search. Stakeholders whose websites did not make reference to ‘forest(s)’, ‘woodland(s)’ and ‘wood(s)’ on any of the three selected webpages (n = 81) were removed from the dataset, leaving 54 stakeholders for further analysis [11]. Although many of the excluded organizations would likely have had some interest in ‘forest(s)’, ‘woodland(s)’ and ‘wood(s)’ they appeared to be of secondary importance to other interests such as conservation and tourism [11].

#### Content analysis level 2

In the second phase of coding, the same web content used in level 1 was further analyzed to classify stakeholders’ specific interests in UK forests. A coding schema for a new set of keywords was developed for this purpose. These were based on the internationally and industry recognized Millennium Ecosystem Assessment [9] classification which was most widely used at the time. The formerly identified 54 stakeholders were further deductively and manually coded and clustered in the three groups, ‘Provisioning Goods and Services’, ‘Regulating Services’ and ‘Cultural Services' [9]. These three groups included the following 29 descriptors as shown in Table 1: ‘Ecosystem services classification’. The careful selection of keywords is vitally important when applying WCM; it is best to use established typologies.

**Table 1:** MEA ecosystem services classification as a descriptor for the content analysis of selected stakeholder webpages.

Provisioning Goods and Services *(products obtained from ecosystems)*	Regulating Services *(benefits obtained from the regulation of ecosystem processes)*	Cultural Services *(non-material benefits obtained from ecosystems)*
Fibre Timber Food Fresh water Fuelwood Biochemicals Genetic resources Ornamental resources	Air quality Climate regulation [global/regional/local] Disease regulation Erosion control Natural hazard regulation Biological control Pollination Water [flood] regulation Water purification Waste treatment	Aesthetic Cultural heritage Education Inspiration Knowledge systems Recreation Tourism Sense of place Social relations Spiritual Religious

Source: based on MEA [9], pp. 56-59 and Raum et al [11].

Serving as keywords, these descriptors were used to identify the specific interests of stakeholders; the stakeholders’ respective webpages (‘home’, ‘about us’, ‘what we do’) were further searched and deductively coded for these keywords as well as their variants. As in the first phase of coding in the keyword search, the parameter for exclusion in the second phase of coding was strictly a nil match result [11].

The full set of criteria used for the verification and the inclusion and exclusion of stakeholders at both analysis level 1 and level 2, are summarized in Table 2. The data relating to each stakeholder was extracted into an Excel spreadsheet for further analysis and categorization. The final results were presented using tables and matrices [11].

**Table 2:** Summary of the verification and inclusion / exclusion criteria on each level.

Inclusion criteria	Exclusion criteria
Verification: • Web presence • Independent organization / stakeholder • Operating in the UK	• No web presence • Part of a larger pre-defined organization • Operating only abroad
Content Analysis Level 1: • Direct reference to ‘forest(s)’, ‘woodland(s)’ and ‘wood(s)’ on selected websites	• No direct reference to ‘forest(s)’, ‘woodland(s)’ and ‘wood(s)’ on selected websites
Content Analysis Level 2: • Direct reference to ecosystem services descriptors stated on selected websites	• No direct reference to ecosystem services descriptors on selected websites

## WCM step‐by‐step guide

**Fig. 3:**
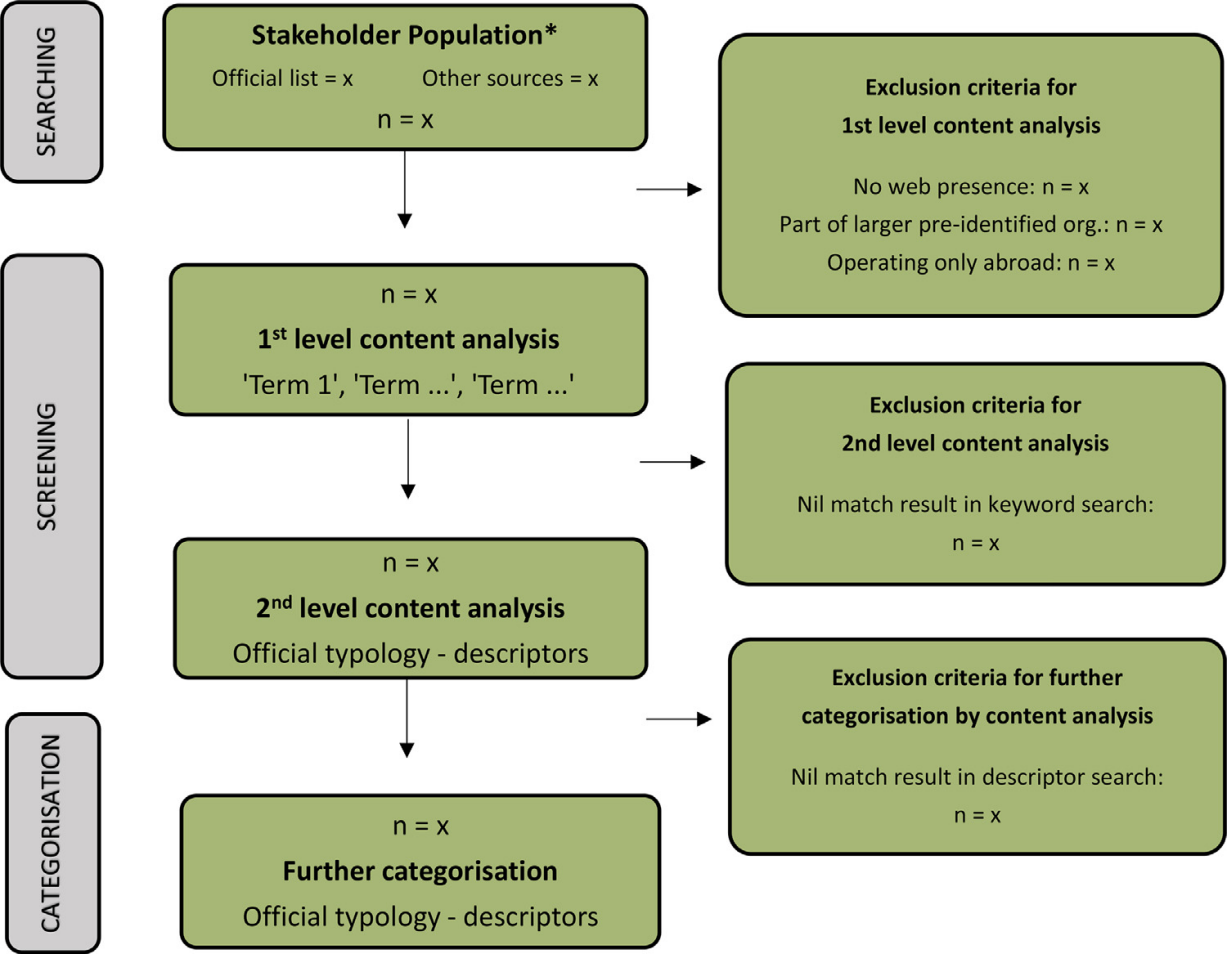
Drawing on Moher's PRISMA standard, this flowchart shows the screening process undertaken to select stakeholders through their webpages as part of WCM. Source: modified from [11]. * Note that in the case of using several existing stakeholder lists, the first step would involve the removal of duplicates.

The general screening process of selected stakeholders is presented in Fig. 3 in form of a flowchart. The flowchart draws on the Preferred Reporting Items for Systematic Reviews and Meta-Analyses (PRISMA) standard [10], considered good practice in systematic reviews in order to perform a transparent document exclusion and inclusion process. For illustration purposes, the flowchart is shown in the context of the UK forestry case study example. If used for other sectors and fields of study, relevant keywords and descriptors will need to be selected for applications in other contexts.

Table 3 provides a summary of the WCM six-step approach, comprising the search, verification, screening, exclusion, categorization, and presentation of results.

**Table 3:** Summary of WCM six-step approach.

**1. Search** 1.1 Determine stakeholders through publicly available government publication / database (can be supplemented by other sources). **2. Ascertain** 2.1 Verify currency of pre-identified stakeholders through an internet search. 2.2 Exclude Apply exclusion criteria (no web presence; part of pre-identified organization; operating only abroad).
**3. Screen (1st- level)** 3.1 Undertake 1st-level content analysis of current stakeholders’ webpages: 1) ‘home’, 2) ‘about us’, and 3) ‘what we do’ (or equivalent) via general sector keyword descriptor (and where applicable, its variants) to verify general stakeholder sector interest. 3.2 Exclude Apply exclusion criteria (nil match result in keyword search). **4. Screen (2nd- level)** 4.1 Undertake 2nd-level content analysis of verified stakeholders’ webpages: 1) ‘home’, 2) ‘about us’, and 3) ‘what we do’ (or equivalent) via specific keyword descriptors derived from an international / industry recognized classification. 4.2 Exclude Apply exclusion criteria (nil match result in keyword search).
**5. Categorize** 5.1 Deductively code remaining stakeholders according to matched keyword descriptors. Undertake further categorization of stakeholders’ three respective webpages via same general and specific keyword descriptors. Deductively code data accordingly.
**6. Present Results** 6.1 Generate WCM-generated tables and matrices.

Source: modified from [11].

## Presentation of findings

Table 4 and 5 provide summary examples of the data obtained through the application of WCM in the context of UK forestry. They illustrate how findings of the stakeholder analysis could be tabulated to provide a broad overview of stakeholders and their interests from a particular sector. It is worthy of note that a key feature of WCM is the facilitation of a clear and concise presentation of results through the generation of tables and matrices.

**Table 4:** A sample outcome of content analysis level 1: Stakeholders with interest in forests in the UK by organizational category.

Governmental Organizations	Commercial Organizations	Not for Profit Organizations	Professional Organizations	Educational / Science Organizations
BEIS DEFRA Forestry Commission …	Acres Wild Woodland Ltd Coppice Resources Ltd. Crops4Energy … …	Ancient Yew Group Community Forests Forest Education Initiative … …	Arboriculture Association Assoc. of National Parks British Horse Loggers … …	Bangor University- School of Natural Sciences – Forestry …

**Source:** modified from [11].

**Table 5:** A sample outcome of content analysis level 2: Stakeholders and their specific interests in forest ecosystem services in the UK grouped according to their interest in the provisioning, regulating and cultural ecosystem services (ES).

National level organizations	Interest in ES		
	Provisioning ES	Regulating ES	Cultural ES
Acres Wild Woodland Ltd			tourism
Association of National Parks			recreation, aesthetics
Bangor University - School of Natural Sciences – Forestry			education
British Horse Loggers	timber		education
Community Forests	fuel wood	water regulation	recreation, education
…	…	…	…

**Source**: modified from [11].

## Limitations of the method

As part of WCM, the verification of stakeholders and their interests is undertaken through the use of a platform which is not subject to independent review. Some websites contain inaccuracies, ill-defined concepts, poorly stated objectives and are designed for marketing, political positioning, or social influencing purposes. Similar to other publicly available information, the data obtained from websites, therefore may, at times, be considered inconsistent, biased or lacking transparency. The ‘green washing’ of companies is a particular case in point. To mitigate uncertainty in the assessment, it is therefore important to use reputable published lists and other authorized sources in the identification of stakeholders as these stakeholders have already been screened for rigor and transparency. Further, for a more detailed analysis, triangulation should be employed with several research methods such as online surveys, questionnaires, interviews, or focus groups [11]. The use of triangulation would allow for direct participation of stakeholders and a more thorough analysis in order to strengthen the research base. This would also help to avoid bias against any stakeholders without a web presence.

## Conclusion

This article introduced WCM, a six-step web content-based method of stakeholder analysis. This systematic and low-cost method can be used to generate basic empirical knowledge about stakeholder interests for use by policy-makers, practitioners, and the scientific community. WCM also allows a certain degree of replicability (within the limits of website changes). WCM is particularly useful for verifying selected stakeholders and for categorizing stakeholders [11]. Since WCM can, by definition, only be used for stakeholders that have a website, triangulation of multiple research methods, such as, for instance, online surveys, questionnaires, interviews, or focus groups, can be applied to avoid bias against stakeholders without a web presence [11]. For illustration purposes, WCM has been introduced in the context of UK forestry. The step-by-step guide, however, can also be applied to other sectors and fields of study and in different countries. In large scale studies, WCM could be modified and used with computer search tools or AI technologies.
